# Characteristics of illegal and legal cigarette packs sold in Guatemala

**DOI:** 10.1186/s12992-016-0219-z

**Published:** 2016-11-25

**Authors:** Rodrigo Arevalo, Juan E. Corral, Diego Monzon, Mira Yoon, Joaquin Barnoya

**Affiliations:** 1 Research Department, Cardiovascular Surgery Unit of Guatemala, 5a Ave 6-22 zona 11, Guatemala City, Guatemala; 2 Department of Medicine, University of Miami Miller School of Medicine and Jackson Memorial Hospital, Miami, FL USA; 3 Division of Public Health Sciences, Department of Surgery, Washington University in St. Louis, School of Medicine, St. Louis, MO USA

**Keywords:** Tobacco industry, Tobacco, Cigarettes, Cigarette packs, Illegal cigarettes

## Abstract

**Background:**

Guatemala, as a party to the Framework Convention on Tobacco Control (FCTC), is required to regulate cigarette packaging and labeling and eliminate illicit tobacco trade. Current packaging and labeling characteristics (of legal and illegal cigarettes) and their compliance with the FCTC is unknown.

**Methods:**

We sought to analyze package and label characteristics of illegal and legal cigarettes sold in Guatemala. We visited the 22 largest traditional markets in the country to purchase illegal cigarettes. All brands registered on tobacco industry websites were purchased as legal cigarettes. Analysis compared labeling characteristics of illegal and legal packs.

**Findings:**

Most (95%) markets and street vendors sold illegal cigarettes; 104 packs were purchased (79 illegal and 25 legal). Ten percent of illegal and none of the legal packs had misleading terms. Half of the illegal packs had a warning label covering 26 to 50% of the pack surface. All legal packs had a label covering 25% of the surface. Illegal packs were more likely to have information on constituents and emissions (85% vs. 45%, *p* < 0.001) and were less expensive than legal ones (USD 0.70 ± 0.7 and 1.9 ± 1.8, *p* < 0.001).

**Conclusions:**

In Guatemala, neither illegal nor legal cigarette packs comply with FCTC labeling mandates. Urgent implementation and enforcement of the FCTC is necessary to halt the tobacco epidemic.

## Findings

### Introduction

Among its marketing practices, the tobacco industry uses the cigarette pack as a way to communicate with current and potential consumers [1]. Shape, size, color, and brand have all been used to increase sales and dismiss consumers’ risk perceptions [1].

Guatemala ratified the WHO Framework Convention for Tobacco Control (FCTC) in 2005 [2]. The FCTC, developed to halt the worldwide tobacco epidemic, mandates countries to ban misleading terms (e.g., light, mild), implement graphic warning labels, and eliminate illicit tobacco trade [2]. Guatemala, as opposed to other Latin American countries, has failed to comply with the FCTC and therefore requires evidence to support policy changes. As a party member, it must include warning labels and pictograms that cover at least 30% of the package [2]. Cigarette packaging and labeling are already included in the Guatemalan legislation. Therefore, tobacco products must include one of five text messages (in Spanish) approved by the Ministry of Health, cover 25% of one side of the pack and include constituents and emissions [3]. In addition, it must take actions aimed at eliminating illicit tobacco trade [2]. Illicit products, including tobacco, do not pay taxes, license agreements, or quality controls. [4–6]. So far, the Guatemalan government has been unable to control illicit tobacco trade. According to the Industry Chamber of Guatemala, illegal cigarettes sales increased from 8.9% of the market share in 2010 to 12% in 2011 [7]. Similar to other countries, inadequate border controls, poor manufacturing surveillance, and ineffective sales controls promote tobacco smuggling [5, 8, 9].

This study sought to evaluate if Guatemala’s legislation complies with the FCTC requirements and to examine if legal and illegal packs comply with Guatemala’s packaging and labeling legislation. Finally, we compared labeling characteristics, price, and country of origin between legal and illegal packs.

## Methods

### Legal packs

Only two companies are registered to manufacture, import and sell cigarettes in Guatemala: Tabacalera Centro Americana, S.A. (TACASA) and Tabacalera Nacional, S.A. (TANASA), subsidiaries of Philip Morris International and British American Tobacco, respectively. All packages are mandated to comply with the national legislation [10, 11].

Legal brands sold by TACASA and TANASA were gathered from the companies’ websites. Then we purchased one pack of each brand at a convenience store in Guatemala City. Given the possibility that legal brands registered in Guatemala can also be smuggled from other countries (e.g., a Marlboro pack smuggled from Honduras), we confirmed the origin of legal packs by looking for the name of TACASA or TANASA in the pack.

### Illegal packs

From May to June of 2012, two trained research assistants purchased cigarettes in 22 traditional markets in the country. Markets were chosen as they are the largest and busiest and therefore where most of the economic activity happens. A two-block radius around each market was also surveyed searching for street vendors selling illegal cigarettes. Staff then asked every store, street vendor, and stall for cigarettes. Points of sale were identified when vendors answered affirmatively to selling cigarettes or when cigarettes were visible anywhere in the store. Afterwards, staff asked for “imported cigarettes” (how vendors refer to illegal cigarettes). One pack of each brand was purchased to get as many brands as possible. An inventory of each brand purchased was kept to avoid duplicates.

### Coding and statistical analysis

A checklist was developed to assess package and label characteristics. Label characteristics included size, pictograms, misleading terms, and language. Price, expiration date, constituents, number of cigarettes per pack, and country of manufacture were also documented.

Percentages and means (standard deviation) were used to summarize data. We performed Chi-square and Analysis of Variance (ANOVA) to compare differences between illegal and legal cigarettes packs. Statistical analyses were done in STATA 11.0.

## Results

A total of 104, 79 illegal (76%) and 25 legal (24%), cigarette packs were purchased. All markets but one sold illegal cigarettes and on average 3.6 different brands were available per market.

Warning label size, “No sales to minors” signage, language, expiration date and price differed between legal and illegal packs (Table 1). All legal packs comply with the warnings as mandated by the national legislation. Overall, 21 illegal packs had no warning label. Illegal packs (85%) were more likely to include information on constituents and emissions compared to legal ones (45%) (*p* < 0.001). The former reported higher concentrations of all three constituents: nicotine, tar and carbon monoxide (Table 1). We found one illegal pack with 10 cigarettes and one legal with 24.

**Table 1:** Characteristics of illegal and legal cigarette packs. Guatemala 2012

	Illegal	Legal	
	79 (76%)	25 (24%)	*p*
Warning label^a^			
No label	21 (27)	0 (0)	0.001
1–25%	24 (30)	25 (100)	
26–50%	32 (40)	0 (0)	
51–75%	2 (3)	0 (0)	
Pictogram	1 (1)	0 (0)	0.01
“No sales to minors” label	64 (81)	25 (100)	<0.001
Missleading terms	8 (10)^b^	0 (0)	0.19
Language			
Spanish	60 (76)	25 (100)	0.005
English	19 (24)	0 (0)	
Expiration date^c^	64 (81)	25 (100)	0.02
Reported constituents (in mg)			
Nicotine	0.97 ± 0.17	0.75 ± 0.11	<0.001
Tar	11.85 ± 1.94	9.5 ± 1.17	<0.001
Carbon monoxide	13.68 ± 3.2	9.9 ± 1.44	<0.001
Price (in USD)	0.7 ± 0.7	1.9 ± 1.8	<0.001

^a^Percentage of the front face covered. Some illegal packs had labels on both sides. No legal packs had labels on both sides

^b^Of these: 6 (8%) “light” and 2 (2%) “ultra light”

^c^Illegal packs were more likely to be expired (45%) compared to legal packs (2%) (*p* < 0.001)

About a third (38%) of illegal packs were manufactured in India, followed by Paraguay (13%), China (13%), and the United States (9%). Manufacturers included Hilton Tobacco PVT. LTD. (member of Bommidala group), Godfrey Phillips India Limited, Tabacalera Hernandarias S.A., Tobacco Hunan Industrial C.O. (subsidiary of China National Tobacco Corporation), China Tobacco Zhejian Industrial C.O., and Vietnam National Tobacco Corporation (state-owned corporation that also manufactures Philip Morris products). Nine packs had no manufacturer information. Legal packs were manufactured in Guatemala by TACASA (12, 48%). TANASA imports cigarettes from Honduras (8, 32%). Both companies import cigarettes from Mexico (TANASA 4, 16% and TACASA 1, 4% respectively).

## Discussion

Guatemala’s current legislation does not comply with the FCTC. Furthermore, neither legal nor illegal cigarette packs comply with the FCTC package and labeling requirements. To our knowledge this is the first study to analyze cigarette pack characteristics and to assess national legislation and compliance with the FCTC in a low/middle income country (LMIC).

Legal packs comply with most of the national legislation [3, 10]. However, they do not comply with the FCTC recommendations on warning labels (more than 30% of surface area and pictograms) [2, 12]. Text warnings are less effective than pictograms in discouraging youth and adults from smoking [13, 14]. This is particularly relevant in a country like Guatemala (and other LMICs) where the illiteracy rate remain high (23.5%) making it even harder to understand text labels [15].

Illegal packs had fewer warning labels and were more likely to include misleading terms. Therefore, illegal packs in most instances do not comply with the national legislation nor the FCTC. Surprisingly, some complied with national legislation and had larger warning labels printed on both sides of the pack. This might be because they were manufactured in a country with legislation different than the Guatemalan and likely to comply with FCTC.

The national legislation mandates that the number of cigarettes per pack is no more, nor less, than 20 [10]. However, we found two noncompliant packs. Regulating the number of cigarettes per pack is also part of the FCTC. Even though the current law complies with the FCTC, it does not include sanctions for manufacturers or sellers and there are no enforcement mechanisms [12, 16]. Therefore, in addition to passing a law, policy makers (in Guatemala and elsewhere) should guarantee that adequate fines are included with the corresponding enforcing mechanisms and responsible agency.

Illegal cigarettes were significantly less expensive than legal ones making them more affordable to low income smokers. They were also readily available in 95% of sampled markets. These markets are usually (21 out of 22) located in rural Guatemala, where most of the poor and indigenous live [17]. Therefore, selling illegal cigarettes in markets is an easy way to reach rural, poor, and indigenous smokers [18].

Our findings should be interpreted in light of some limitations. In Guatemala only TACASA and TANASA are registered to manufacture, import, and sell cigarettes. Packs do not have a tax stamp nor a license is required to sell tobacco. Therefore, we had to rely on the list available from the companies’ website to classify cigarettes as legal or illegal. We could not verify if some of the packs classified as legal were not just high quality counterfeits. Even though the largest markets in the country were surveyed, we recognize that given the size of the informal economy, it is impossible to document all illegal cigarettes. Street vendors are mobile and there might be other sources for illegal cigarettes. Regardless, these data represent a first attempt to characterize cigarette packs in a LMIC and document the low price of cigarettes (legal and illegal) in a country with poor FCTC implementation. Consequently, policy makers and healthcare advocates need to review and update the current law to meet the FCTC requirements on labeling and smuggling.

## Conclusions

Cigarette packaging and labeling in Guatemala are neither compliant with the national legislation nor with the FCTC. The lack of a clear definition of legal cigarettes is just another hurdle to control smuggling. Mandating a tax stamp in packs and requiring a license to sell tobacco might contribute to control smuggling and should be explored by policy makers in Guatemala and elsewhere. In addition to raising the price of cigarettes, this would provide a mechanism to easily differentiate legal from illegal cigarettes and to control their availability in the informal economy.

## Abbreviations

ANOVA: Analysis of variance
FCTC: Framework convention on tobacco control
LMIC: Low/middle income country
TACASA: Tabacalera Centro Americana, S.A.
TANASA: Tabacalera Nacional, S.A.
